# Near-Infrared Spectroscopy based Neurofeedback of Prefrontal Cortex Activity: A Proof-of-Concept Study

**DOI:** 10.3389/fnhum.2016.00633

**Published:** 2016-12-16

**Authors:** Beatrix Barth, Ute Strehl, Andreas J. Fallgatter, Ann-Christine Ehlis

**Affiliations:** ^1^Psychophysiology and Optical Imaging, Department of Psychiatry and Psychotherapy, University of TuebingenTuebingen, Germany; ^2^Graduate School of Neural and Behavioral Sciences, University of TuebingenTuebingen, Germany; ^3^Institute for Medical Psychology and Behavioural Neurobiology, University of TuebingenTuebingen, Germany; ^4^Werner Reichardt Centre for Neuroscience, University of TuebingenTuebingen, Germany; ^5^Graduate School LEAD, University of TuebingenTuebingen, Germany

**Keywords:** near-infrared spectroscopy, NIRS neurofeedback, prefrontal cortex, healthy, learning effects

## Abstract

Neurofeedback is a promising tool for treatment and rehabilitation of several patient groups. In this proof of principle study, near-infrared spectroscopy (NIRS) based neurofeedback of frontal cortical areas was investigated in healthy adults. Main aims were the assessment of learning, the effects on performance in a working memory (n-back) task and the impact of applied strategies on regulation. 13 healthy participants underwent eight sessions of NIRS based neurofeedback within 2 weeks to learn to voluntarily up-regulate hemodynamic activity in prefrontal areas. An n-back task in pre-/post measurements was used to monitor neurocognitive changes. Mean oxygenated hemoglobin (O_2_Hb) amplitudes over the course of the sessions as well as during the n-back task were evaluated. 12 out of 13 participants were able to regulate their frontal hemodynamic response via NIRS neurofeedback. However, no systematic learning effects were observed in frontal O_2_Hb amplitudes over the training course in our healthy sample. We found an impact of applied strategies in only 5 out of 13 subjects. Regarding the n-back task, neurofeedback appeared to induce more focused and specific brain activation compared to pre-training measurement. NIRS based neurofeedback is a feasible and potentially effective method, with an impact on activation patterns in a working memory task. Ceiling effects might explain the lack of a systematic learning pattern in healthy subjects. Clinical studies are needed to show effects in patients exhibiting pathological deviations in prefrontal function.

## Introduction

Modern neuroimaging methods offer the opportunity to feedback aspects of brain physiology, so that people can learn to voluntarily regulate and modify them. This skill, in turn, can be a useful tool in treatment or rehabilitation by directly targeting neurophysiological deficits (Bartholdy et al., 2013; Holtmann et al., 2014; Kim and Birbaumer, 2014). The process of continuously providing feedback of physiological signals of a person’s brain – and thereby giving them the means to learn to control them – is known as neurofeedback.

Neurofeedback is mostly hypothesized to be based on an operant conditioning process comprising reinforcements for desired brain states and thereby leading to conscious self-regulation of brain activity. However, not only operant conditioning (i.e., trial and error) is decisive for learning brain self-regulation, but also classical conditioning processes (i.e., transfer into everyday life), the two-process-theory (i.e., identifying strategies; associating feedback with interoceptive stimuli) as well as individual motivational aspects play a critical role (Strehl, 2014). Several recent studies have been carried out on EEG feedback, and clinical improvements could repeatedly be shown. Hereinafter, only a few publications are mentioned. EEG neurofeedback was found to successfully help reduce seizures in epilepsy (Rockstroh et al., 1993; Kotchoubey et al., 2001) and abate core symptoms of ADHD (Arns et al., 2014). In criminal psychopaths, reduced aggression, impulsivity, and behavioral approach tendencies could be observed after an EEG neurofeedback intervention (Konicar et al., 2015). Beyond that, studies on EEG neurofeedback in healthy participants also provide evidence of beneficial outcomes and cognitive or affective gains (Gruzelier, 2014).

So far, only a few EEG feedback studies distinguished participants that learned brain self-regulation from participants that did not. According to these few studies, around 50% of participants are successful in modulating electrophysiological brain activity and show different training outcomes compared to less successful participants (Gruzelier et al., 1999; Hanslmayr et al., 2005; Doehnert et al., 2008; Weber et al., 2011). Up till now, however, to our knowledge no study about detailed learning processes and learning types over the training course has been published.

Compared to EEG, functional imaging methods such as fMRI have the advantage of a higher spatial resolution. In several investigations, it was shown that healthy participants are able to learn brain self-regulation based on BOLD feedback within one to 10 sessions (Weiskopf, 2012; Sulzer et al., 2013). Similar to fMRI, NIRS also measures local changes in blood metabolism, and therefore comparable results can be expected for NIRS neurofeedback. Compared to fMRI, NIRS offers several advantages: fMRI measurements are expensive and locally bound to the place where the scanner is installed. Furthermore, fMRI has strict limitations in study design as participants have to stay in an immobile, reclined position due to the high sensitivity to movement artifacts. Finally, the noise in the scanner also must be taken into account as a disadvantage of fMRI. In contrast to that, NIRS is relatively insensitive to motion artifacts and measurements can be conducted in a more natural environment (Obrig, 2014). Preparation for measurements can be carried out fast, also compared to EEG. Thus, NIRS should be considered an alternative neurofeedback method particularly for psychiatric patients.

Previous studies indicate that NIRS is a suitable method to assess, amongst others, different levels of cognitive load, and the prefrontal cortex (PFC) has been shown to be involved in the processing of working memory load (Ayaz et al., 2012; Fishburn et al., 2014; Herff et al., 2014). N-back paradigms are convenient tasks to generate differently demanding working memory load conditions. During n-back tasks, participants are instructed to perpetually keep in mind either the last (lower working memory load), the penultimate (higher working memory load) or more items changing in quick succession. In this context, NIRS signals have been shown to be sensitive to working memory load specifically within the left inferior frontal gyrus (Ayaz et al., 2012). These findings have been substantiated by another study revealing a linear increase in bilateral PFC activation with increasing working memory load in an n-back task (Fishburn et al., 2014). Likewise, recent evidence indicated that hemodynamic responses in the PFC as assessed by NIRS can be applied to classify different levels of working memory load induced by an n-back task (Herff et al., 2014). Altogether, these results provide evidence that NIRS is a suitable method to assess cognitive load.

Up till now, however, only a few studies have employed NIRS neurofeedback protocols. One study investigated a NIRS based feedback regulated BCI measuring prefrontal activity in healthy adults (Ayaz et al., 2009). BCIs offer the possibility of communication between the brain and an external apparatus. In this context, the study revealed significant differences in mean oxygenation changes during task versus rest period and furthermore provided evidence indicating learning and adaptation processes over two training days. Three studies investigated motor cortex activation changes combining a motor imagery task with NIRS neurofeedback (Kanoh et al., 2011; Mihara et al., 2012; Kober et al., 2014). Here, over the training course the signal-to-noise ratio of the O_2_Hb signal increased significantly. A pilot study aimed to investigate NIRS neurofeedback of O_2_Hb within the PFC as a treatment method for children with ADHD (Marx et al., 2014). After 12 sessions of NIRS feedback, ADHD symptoms decreased significantly.

Despite the few studies that provide evidence for the feasibility of NIRS based neurofeedback, learning processes during the training as well as the underlying mechanisms remain largely unexplored. However, these are decisive components that provide the basis for an optimal implementation of feedback designs in clinical practice (e.g., regarding the number of sessions, modality of feedback, instructions, etc.). Therefore, the present proof of principle study aimed at further investigating NIRS based prefrontal neurofeedback in a healthy adult sample over eight training sessions. The number of training sessions was set based on the number of sessions conducted in previous NIRS based feedback studies in healthy subjects ranging from one to eight. To optimally describe learning processes, we selected the upper bound of sessions conducted, i.e., eight sessions. The main objective was to describe learning patterns. In addition, the effect of individual regulation strategies on regulation performance as well as neurocognitive changes induced by the neurofeedback training in a working memory task (n-back) were analyzed.

## Materials and Methods

### Participants

Thirteen healthy subjects (eight female; five male) completed eight training sessions [mean age: 28 (*SD* = 4.51) years]. All participants had normal or corrected-to-normal vision. Two of the subjects were left-handed, the other 11 were right-handed. No participant had a known history of any neurological or psychiatric disorder (based on self-report). The highest achieved education level was a doctoral degree for two of the subjects, an academic degree for nine participants and a vocational education for the remaining two. All subjects gave written consent to the study in accordance with the informed consent regulations of the institution where the research was conducted. The newly established frontal lobe focused, NIRS based neurofeedback training was approved by the ethics committee at the University Tübingen and University Hospital Tübingen for studies in both patients with ADHD (297/08; 434/2010BO1) and healthy participants (018/2015BO2); the data reported here were derived from the first pilot trainings conducted in healthy subjects.

### Procedure

#### Working Memory Task (n-Back)

Prior to the first training session as well as after completion of eight sessions, a working memory (n-back) task was conducted to investigate activation changes induced by prefrontal NIRS neurofeedback. As already mentioned above, previous studies have shown that the PFC plays a critical role in working memory function (Baddeley, 2003; Owen et al., 2005).

**FIGURE 1:**
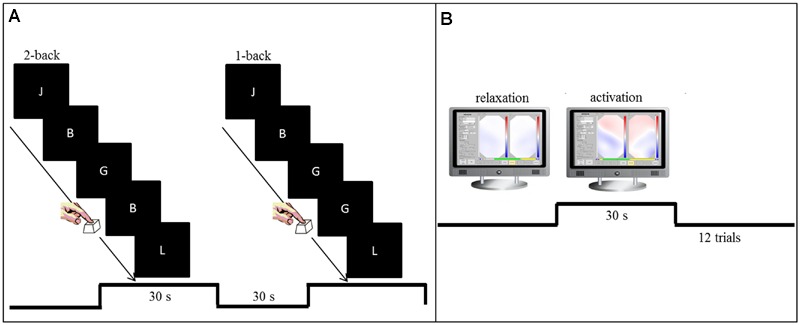
**(A)** Setup for the pre-training and post-training working memory task (n-back). In the 2-back condition (high working memory load), participants were asked to press the space bar as fast as possible whenever the presented letter was the same as the penultimate letter. During the 1-back condition (low working memory load), subjects were instructed to press the space bar as fast as possible whenever a letter was identical to the preceding letter. **(B)** Training setup for the NIRS based neurofeedback sessions. Visual feedback of O_2_Hb changes over left and right prefrontal and temporal sites was presented as topographic 2D view on a computer screen. The signal turned more reddish whenever O_2_Hb increased or more bluish whenever a decrease in O_2_Hb occurred. Participants were asked to focus on the frontal part of the depicted head.

The task comprised two conditions of a verbal n-back paradigm in which white letters (“A,” “B,” “C,” “D,” “E,” “F,” “G,” “H,” “J,” “L”) were presented in pseudo-randomized order in quick succession against a black background on a computer screen (**Figure 1A**). The measurement started with a 10-s baseline period. In the 2-back condition (high working memory load), participants were asked to press the space bar as fast as possible whenever the presented letter was the same as the penultimate letter. During the 1-back condition (low working memory load), subjects were instructed to press the space bar as fast as possible whenever a letter was identical to the preceding letter. Letters were presented in pseudo-randomized order with a presentation time of 300 ms and an interstimulus interval of 1700 ms resulting in 20-s task segments. The two conditions were carried out in an alternate block-wise fashion. Each task block was followed by a 20-s period of rest during which participants were asked to remain seated without moving. Each condition was repeated three times. A total of 12 targets were presented across task segments for all conditions.

#### NIRS Neurofeedback

Subjects were seated in front of a monitor at a distance of approximately 80 cm in a completely dark and sound-attenuated room. Eight NIRS feedback sessions were conducted, each on a different day within 2 weeks. Each session lasted approximately 30 min including preparation time with 12 min feedback training. Any training started with a 15-s baseline measurement and comprised in total 12 trials of activation separated by trials of relaxation. Each trial lasted 30 s.

Via headphones, participants were presented with a higher pitch tone (1000 Hz; 100 ms duration) that served as call to start up-regulation. A deeper pitch tone (450 Hz; 100 ms) signaled the end of the up-regulation trial and contiguously the beginning of relaxation for the next 30 s up to the next activation trial. Sounds were presented by means of Presentation Version 14.5 (Neurobehavioral Systems, Inc.). Participants continuously received visual feedback on a screen that directly mapped their hemodynamic response in all channels in a superior 2D view of a simplified brain (**Figure 1B**). All participants received the same color range for visual feedback relative to their respective baseline recorded at the beginning of each session. The signal turned more reddish whenever there was an increase in O_2_Hb or more bluish whenever a decrease in O_2_Hb occurred. Participants were instructed to try to turn the frontal part of the depicted head as red as possible whenever they heard the higher pitch (activation) and whenever they heard the deeper pitch they were instructed to let the head turn blue again (relaxation). Participants were explicitly asked to focus only on the frontal part of the depicted head. Furthermore, they were instructed to sit as still as possible. Right after each session, subjects were asked to write down the strategies they had applied.

### NIRS Recordings and Analysis

Relative level changes of O_2_Hb and HHb over frontal sites during activation phases were measured by means of the ETG-4000 continuous wave system (Hitachi Medical, Co., Japan). The ETG-4000 uses light of two different wavelengths (695 ± 20 nm and 830 ± 20 nm) whose frequencies are modulated for each wavelength and channel, respectively (Plichta et al., 2006). All concentration changes of O_2_Hb and HHb depend on the path length of the NIR light in the brain as the optical path length cannot be measured by means of continuous wave systems. Therefore, the hemoglobin quantity is scaled in molar concentration multiplied by the unknown path length (mmol × mm).

**FIGURE 2:**
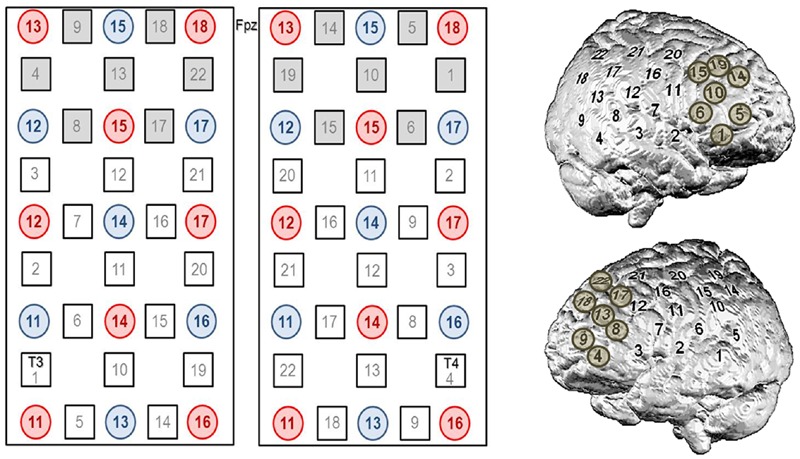
**Channel configuration of the 3 × 5 optode probesets consisting of seven photo detectors (blue) and eight light emitters (red), respectively, resulting in 22 channels per probeset and a total of 44 channels.** In accordance to the international 10–20 system of electrode placement (Jasper, 1958), we used Fpz as mid-point and additionally T3 and T4, respectively, as marker positions to place the rearmost channel in the lowest row of both probesets on each side. The seven most frontally located channels on each side (i.e., #4, 8, 9, 13, 17, 18, 22 on the left and #1, 5, 6, 10, 14, 15, 19 on the right) were defined as ROI channels for neurofeedback analyses.

To cover frontal sites on both hemispheres appropriately, we used two 3 × 5 probesets (consisting of seven photo detectors and eight light emitters, respectively) resulting in 22 channels per probeset and a total of 44 channels (**Figure 2**). The interoptode distance was 30 mm and the sampling rate was set to 10 Hz. In accordance to the international 10–20 system of electrode placement (Jasper, 1958), we used Fpz as mid-point and additionally T3 and T4, respectively, as marker positions to place the rearmost channel in the lowest row of both probesets on each side. By means of this spatial information, positions of optodes on the head-surface were used for the probabilistic registration of NIRS channel positions onto a standard brain and MNI coordinates (Tsuzuki et al., 2007) with reference to the macroanatomical brain atlas LBPA40 (Shattuck et al., 2008) providing a probabilistic labeling of brain regions.

For the analysis of the regulation performance, the seven most frontally located channels on each side (i.e., # 4, 8, 9, 13, 17, 18, 22 on the left and # 1, 5, 6, 10, 14, 15, 19 on the right) were defined as ROI channels. For the n-back task, we intended to get a more general overview of activation changes induced by the training also beyond prefrontal regions and therefore no ROI channels were defined for further analyses.

As part of oﬄine data analysis and preprocessing, the NIRS raw signal was filtered with a 0.01 Hz high pass filter and a 0.3 Hz low pass filter and manually corrected for artifacts (rejection by visual inspection). For further analysis, we focused only on changes in O_2_Hb over ROI channels of both hemispheres. The task-related O_2_Hb concentration changes were referred to a 2-s baseline interval prior to the regulation phase. Finally, O_2_Hb amplitudes during the regulation phase were averaged for each session and participant and used to evaluate regulation performance.

### Statistical Analysis

The present study involved qualitative (applied strategies and performance, self-regulation in PFC during NIRS neurofeedback) as well as quantitative (working memory task) questions and analyses. MATLAB R2012b (The MathWorks, Natick, MA, USA) was used for preprocessing of NIRS data, IBM SPSS Statistics 22 (Armonk, NY, USA) was utilized for all statistical analyses. Data were tested for normal distribution with the Kolmogorov–Smirnov test. For analyses of neurofeedback data, O_2_Hb amplitudes were compared against zero for each session for frontal ROI channels using one-sided Student’s *t*-test. For analyses of n-back data, O_2_Hb changes were compared for 2-back versus 1-back as well as post-training versus pre-training in the n-back task for each channel using paired Student’s *t*-test. For n-back data, all reported *p*-values are based on two-sided significance tests due to the exploratory character of the study and as the direction of changes was not clear beforehand. The significance level was set to 0.05 and adjusted for multiple comparisons according to the Bonferroni–Holm correction. Additionally, effect sizes (Cohen’s *d*) are reported (Cohen, 1988).

## Results

### Applied Strategies and Performance (Qualitative Analysis)

**FIGURE 3:**
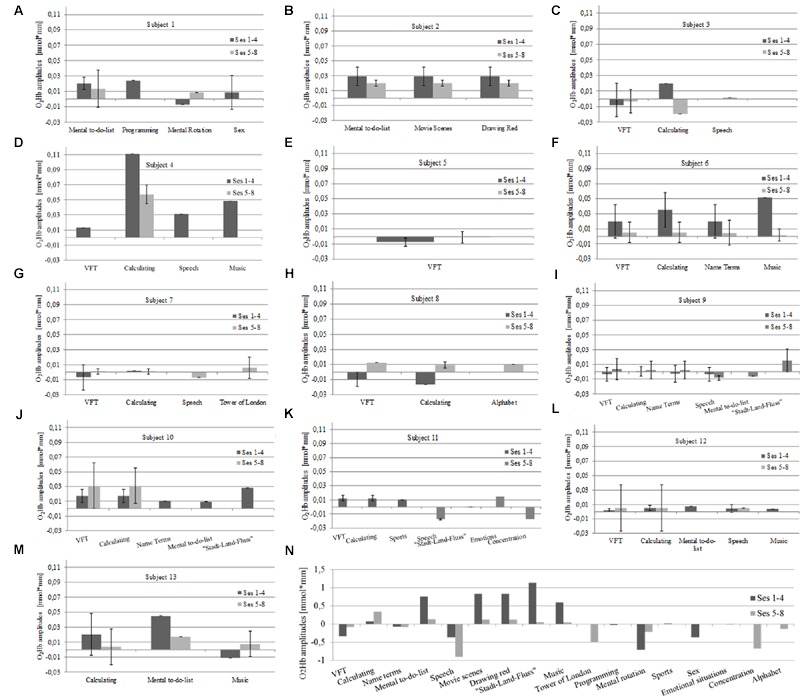
**Interrelation between strategy and mean O_2_Hb amplitudes in left prefrontal channels over the course of eight NIRS based neurofeedback sessions in each individual subject on a descriptive level **(A–M)**.** O_2_Hb amplitudes are exemplarily illustrated only for the left hemisphere as amplitudes on the right hemisphere exhibited similar patterns. For qualitative analysis on a descriptive level of interrelations between amplitude and strategy at the group level, amplitudes were z-transformed **(N)**. VFT, verbal fluency task.

As for the qualitative analysis aiming to describe regulation strategies and associated performance, we looked at O_2_Hb amplitudes of the seven foremost channels in both optode arrays for each session on a descriptive level. First, we looked at potential systematic relations between performance and applied strategies for up-regulation in each session for each participant. We found systematic influences of the applied strategies (**Table 1**) on performance only in five subjects (i.e., subjects 1, 3, 4, 10, 13). In these participants, some of the strategies seemed to induce higher amplitudes compared to the other strategies. For example, looking at **Figure 3M**, the strategy “mental to-do-list” is associated with markedly higher amplitudes as compared to the remaining two strategies. However, for the other eight participants we did not find any systematic impact of strategies such as verbal fluency tasks or mental calculations on amplitude size. For example, **Figure 3H** reveals comparable amplitudes for all applied strategies (i.e., verbal fluency task, calculating, reciting the alphabet) over the training course. Also, overall, most approaches seemed to be generally suited to induce activation of the PFC and strategies did not tend to change over the training course (**Figures 3A–M**). For qualitative analysis (i.e., on a descriptive level) at the group level, we used z-transformed mean amplitudes. At the group level, the most successful strategies seemed to be “Stadt-Land-Fluss” (a game in which cities, countries, rivers and terms of other categories with the same first letter have to be found), mental to-do lists, imagination of movie scenes and “drawing red.” These strategies were superior to the remaining ones mainly for the first part of the training process whereas this superiority diminished in the last four sessions (**Figure 3N**).

**Table 1:** Regulation strategies applied by subjects over the course of the eight training sessions.

Strategy	Ses 1	Ses 2	Ses 3	Ses 4	Ses 5	Ses 6	Ses 7	Ses 8
Verbal Fluency Task	7	6	8	8	5	6	5	8
Calculating	5	6	6	2	7	5	7	6
Name terms of certain categories (e.g., cities, countries, animals)	2	3	2	4	4	0	4	3
Mental to-do-list	2	1	1	3	5	1	3	2
Speech associated strategies (conjugating Latin verbs, inner speech, backward spelling)	2	3	1	2	2	2	1	2
Imagination of movie scenes	1	1	1	1	1	1	1	1
Drawing red	1	1	1	1	1	1	1	1
“Stadt-Land-Fluss”	0	0	1	0	0	2	2	0
Music associated strategies (imagine playing the piano, singing, arranging chords)	1	0	1	1	1	0	1	0
Tower of London	0	0	0	0	0	1	1	1
Programming	0	1	1	0	0	0	0	0
Mental rotation	0	0	0	1	0	1	0	0
Sport movements	1	1	0	0	0	0	0	0
Thinking about sex	0	1	0	1	0	0	0	0
Imagination of emotional situations	0	0	0	0	1	1	0	0
Concentration	0	0	0	0	0	0	1	0
Reciting the alphabet	0	0	0	0	0	1	0	0

*Numbers indicate how many of the participants resorted to the respective strategy for each session.*

**FIGURE 4:**
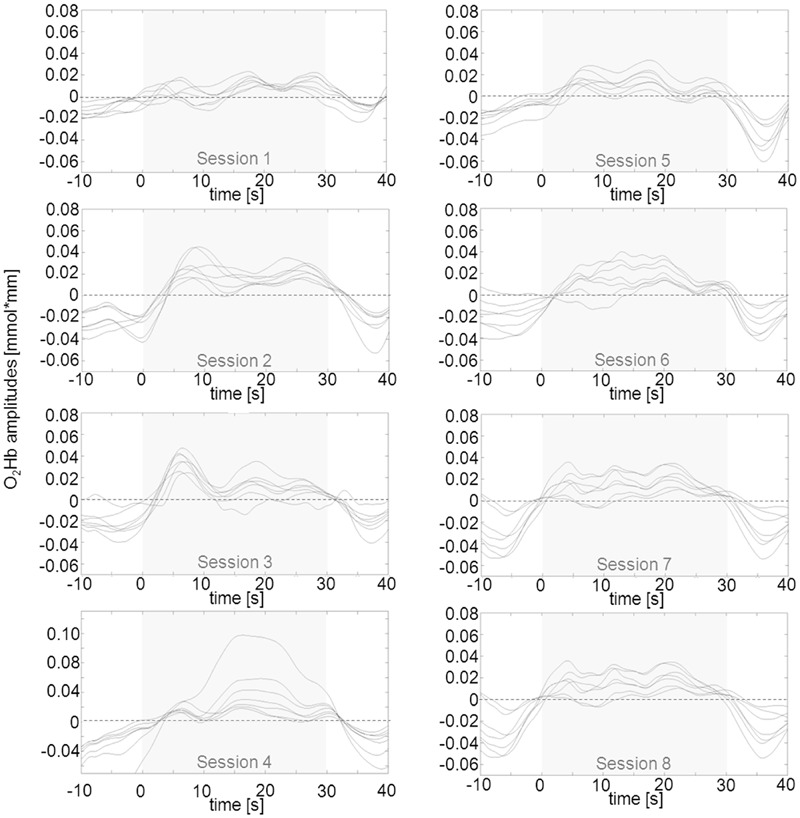
**Qualitative data analysis of mean O_2_Hb amplitudes in left prefrontal channels over the course of eight NIRS based neurofeedback sessions in one exemplary participant (subject #2) out of five that were able to voluntarily up-regulate over the whole training course.** The part shaded in gray represents the upregulation phase of 30 s duration. O_2_Hb concentration changes on the right were similar but marginally less pronounced.

**FIGURE 5:**
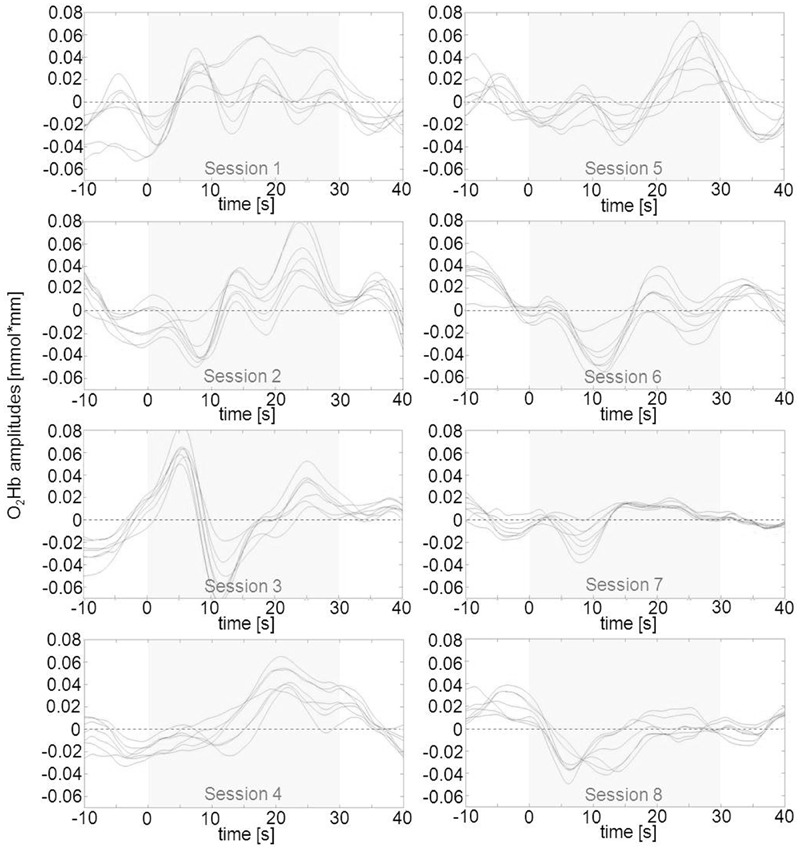
**Qualitative data analysis of mean O_2_Hb amplitudes in left prefrontal channels over the course of eight NIRS based neurofeedback sessions in one exemplary participant (subject #13) out of two that were able to voluntarily up-regulate activity during the second half of the trials of each session.** The part shaded in gray represents the upregulation phase of 30 s duration. Changes in O_2_Hb concentration on right prefrontal sites were similar but marginally less pronounced.

**FIGURE 6:**
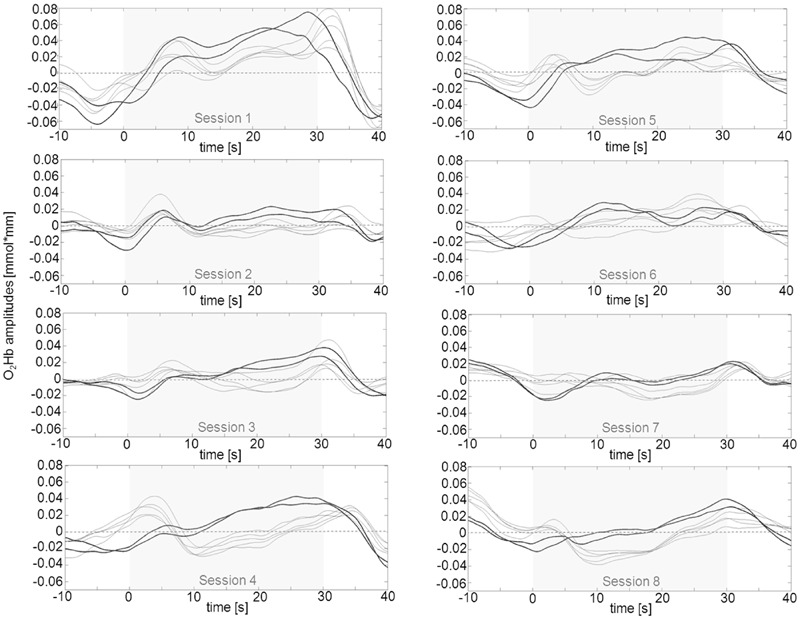
**Qualitative data analysis of mean O_2_Hb amplitudes in left prefrontal channels over the course of eight NIRS based neurofeedback sessions in one exemplary participant (subject #6) out of two that were able to voluntarily up-regulate activity over the whole training course, but in only two neighboring prefrontal channels at a time (bold lines representing channels #8 and 9 for the first session, #4 and 9 for the second session and #4 and 8 for the remaining sessions).** These pairs of channels exhibit more pronounced positive amplitudes as compared to the other frontal ROI channels. Changes in O_2_Hb concentration occurred over virtually identically located neighboring channels in both hemispheres. The part shaded in gray represents the regulation phase of 30 s duration.

In a second step, we tried to classify different types of learning patterns which resulted in five categories. Five participants were able to up-regulate from the first session over the whole training course (**Figure 4**). Two subjects were able to up-regulate from the first session over the whole training course but only for the second half of each regulation trial (**Figure 5**). Two participants were able to up-regulate from the first session over the whole training course, but in only very small parts of the frontal cortex at a time (always just two neighboring channels; **Figure 6**). For three participants, voluntary up-regulation occurred rather irregularly from session to session, but all of them were successful in at least four sessions. Only one person did not learn voluntary activation of PFC at all.

### Self-Regulation in Prefrontal Cortex during NIRS Neurofeedback (O_2_Hb Amplitudes)

Twelve out of 13 participants were able to up-regulate prefrontal activity by means of NIRS based neurofeedback training. However, over the training course no systematic increase in O_2_Hb amplitudes could be observed (**Table 2**). Instead, mean amplitudes seemed to fluctuate from session to session. In the second to last session (i.e., session #7), a strong decline of performance occurred. This temporary decline might be due to a loss of motivation near completion of the training which wears off again in the final session. Except for session #7, mean amplitudes of the total sample exhibited values above zero (**Table 2**) which shows a continuously successful regulation performance. These positive amplitude values are significantly different from zero for sessions #1, 2, 5, and 6 on the left side after correction for multiple testing (**Table 2**). However, on the right side, no significant differences are only observable after correction for multiple comparisons (**Table 2**). Hence, we can conclude that the present sample of healthy subjects performed on a high level from the beginning with probably only little scope left for further learning effects.

**Table 2:** Mean O_2_Hb amplitudes in left and right prefrontal channels over the course of eight NIRS based neurofeedback sessions as well as results of one-sided one sample *t*-test comparing amplitude size for each probeset and session against zero.

SES	Mean amplitudes [mmol^∗^mm] (*SD*)	One sample *t*-test probeset (left) *t* (*p*)	One sample *t*-test probeset (right) *t* (*p*)
1	0.0116 (0.0228)	2.19 (0.025) ^∗^	1.38 (0.097)
2	0.0249 (0.0293)	3.80 (0.002) ^∗^	2.30 (0.020)
3	0.0054 (0.0200)	1.28 (0.113)	0.62 (0.273)
4	0.0066 (0.0192)	1.88 (0.043)	0.41 (0.345)
5	0.0164 (0.0176)	4.19 (<0.001) ^∗^	2.24 (0.023)
6	0.0134 (0.0228)	2.82 (0.008) ^∗^	1.31 (0.107)
7	-0.0022 (0.0227)	0.51 (0.310)	-1.01 (0.166)
8	0.0096 (0.0251)	2.04 (0.032)	0.79 (0.223)

*^∗^indicates significant effects after correction for multiple comparison.*

### Working Memory (n-Back) Task

**FIGURE 7:**
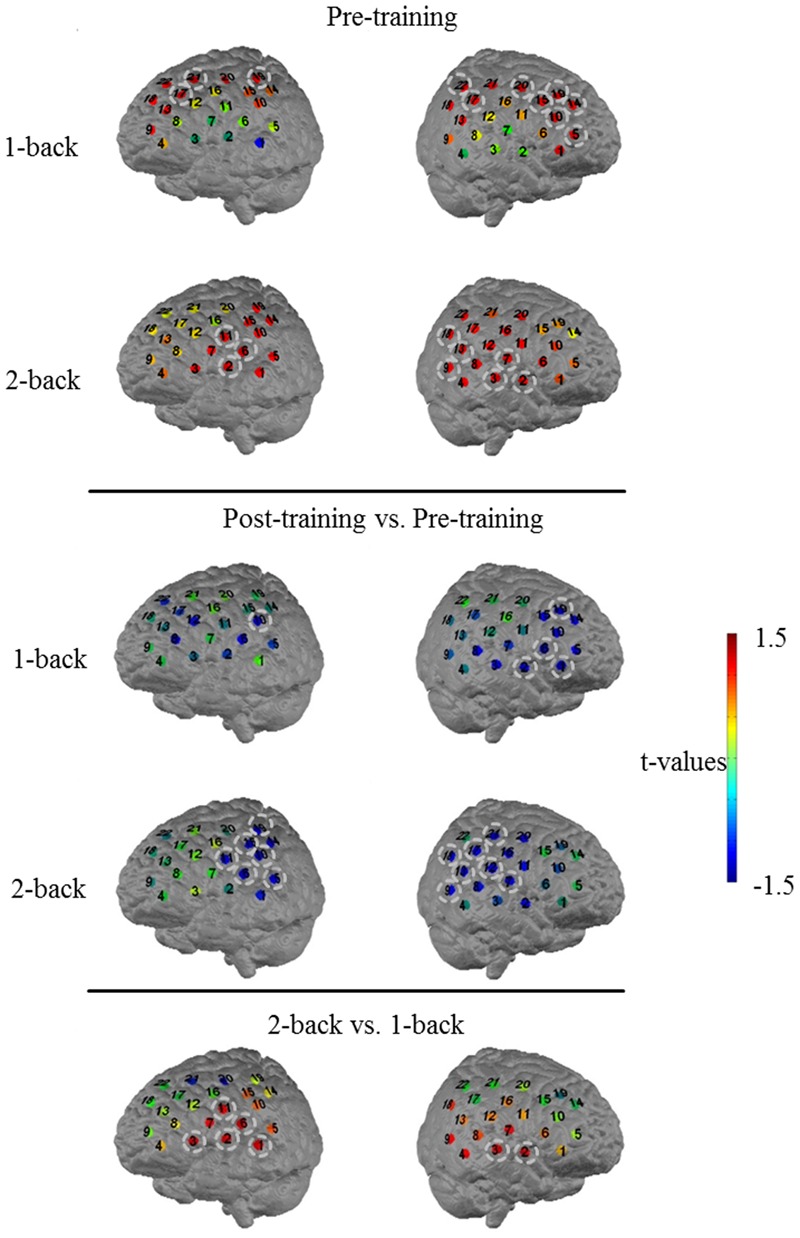
***T*-maps for the comparison of O_2_Hb concentration changes during 1-back at pre-training (first row) and during 2-back at pre-training (second row).** Further *t*-maps for the comparison of O_2_Hb concentration changes during 1-back at post-training versus pre-training (third row) and during 2-back at post-training versus pre-training (fourth row) as well as for the comparison of pre-traininig O_2_Hb concentration differences between 2-back and 1-back (fifth row). Numbers indicate the 22 channels on each channel array. Channels highlighted by circles indicate channels with non-significant differences after Bonferroni–Holm correction but effect sizes ≥ 0.5. For illustration purposes, the probesets are mapped on a standard brain.

In a first, general step of the analysis, we compared the 1-back and 2-back condition of the working memory task for the pre-training measurement with respect to task-related changes in O_2_Hb concentration (**Figure 7**) in order to depict general activation patterns associated with the differences in working memory load. T-maps of this contrast indicated increased activation in language associated areas in the 2-back compared to the 1-back condition on both hemispheres (left: channels #1, 2, 3, 6, and 11 covering middle temporal gyrus, pars triangularis, superior temporal gyrus and pre-motor cortex, 1.92 ≤*t*22 ≤ 2.50, 0.02 < *p* < 0.08, 0.5 < *d* < 0.7; right: channels #2 and #3 covering middle temporal gyrus and temporopolar area, *t*22 = 2.34 and 1.85, *p* = 0.04 and 0.09, *d* = 0.6 and 0.5, respectively) that did – however – not survive a Bonferroni–Holm correction despite medium effect sizes.

In a second step, to investigate task-related changes in O_2_Hb concentration potentially induced by NIRS neurofeedback, we compared activation patterns of pre-training data to post-training data in 1-back and 2-back, respectively. For the low cognitive load condition (1-back), after Bonferroni–Holm correction, a statistically non-significant decrease in O_2_Hb concentration in post- compared to pre- assessment was solely present in single channels located in frontal and temporal parts of both channel arrays (channel #10 on the left covering primary somatosensory cortex and channel #1, 2, 6, 19 on the right side of the head covering temporopolar area, pars triangularis, and dorsolateral PFC, *t*22 = -1.93 and -2.28, -1.89, -1.88, -1.98, respectively; 0.04 < *p* < 0.08; -0.6 < *d* < -0.4; see **Figure 7** for non-significant decrease in O_2_Hb concentration). For the high cognitive load condition (2-back), differences between the two assessments were more apparent and occurred predominantly in temporal parts of the measurement array (**Figure 7**; decrease in O_2_Hb concentration for channels #5, 6, 10, 11, 15, and 19 on the left, and for channels #7, 9, 12, 13, 16, 17, 18, and 21 on the right side, -2.58 ≤*t*22 ≤-2.01 and -2.78 ≤*t*22 ≤-1.84, respectively; 0.01 < *p* < 0.07 and -0.7 < *d* < -0.6), covering left superior temporal gyrus, primary somatosensory cortex, pre-motor cortex as well as supramarginal gyrus and right subcentral area, fusiform gyrus as well as parts of Wernicke’s area. However, again, these effects did not withstand a Bonferroni–Holm correction for multiple statistical comparisons despite medium to high effect sizes.

To sum up these data, participants showed rather widespread activation patterns over frontal, middle and temporal sites (1-back) and temporally located language associated areas (2-back) during pre-training assessments as compared to post-training assessments. In the follow-up measurement, they tended to show decreased activation in frontal (1-back), middle (1-back), and temporal (1- and 2-back) areas with medium effect sizes. All channels with tendential changes were adjacent channels and the observed differences occurred in both hemispheres, even though the effects seemed to be slightly more pronounced in the left hemisphere.

## Discussion

This proof of principle study aimed to unravel learning processes, the impact of strategies on individual learning and regulation as well as different learning patterns in NIRS neurofeedback. Studies on NIRS neurofeedback are scarce (Kanoh et al., 2011; Mihara et al., 2012; Kober et al., 2014; Marx et al., 2014), and up till now only one pilot study has examined NIRS feedback in patients, namely children with ADHD (Marx et al., 2014).

Our results confirm that it is possible to control hemodynamic responses in prefrontal brain areas even over the course of only a few training sessions of NIRS feedback for healthy subjects. 12 out of 13 participants were able to voluntarily activate their PFC with the help of NIRS feedback. The data suggest slightly more pronounced regulation ability in the left hemisphere which might be due to the high amount of verbal strategies applied by our sample. Beyond that, we could not reveal any immediate impact of applied strategies on regulation performance for the individual sessions. All approaches appeared to be effective to achieve prefrontal up-regulation, and overall participants tended to adhere to their chosen strategies over the training course. The stability of regulation approaches right from the first session might be due to the fact that these strategies already were well-elaborated and therefore worked almost from the beginning. For example, only to mention one of the regulation approaches, verbal fluency tasks have been shown to induce increased activation in prefrontal areas (Frith et al., 1991; Schlösser et al., 1998).

In line with this relative stability of regulation strategies across sessions, prefrontal O_2_Hb amplitudes did not systematically increase over the training period. This might be due to ceiling effects that in turn might be ascribed to our selective sample consisting of psychologists, physicians, physicists, and other professional groups that due to their educational level and field of professional activity already might have an above-average frontal control (Vakhtin et al., 2014) and/or prior knowledge about prefrontal activation strategies. Furthermore, visual feedback in the current training setup was rather unspecific: the feedback layout of hemodynamic activity as target parameter for regulation was a plain visualization of concentration changes of O_2_Hb on a simplified animated topographic 2D view, also including irrelevant channels in more posterior areas. Moreover, there was no correction for general arousal which makes it harder to specifically self-regulate frontal brain activity without inducing widespread activation changes all over the brain. Taken together, these aspects might explain the lack of a systematic learning effect over the training course. In this regard, to substantiate participants’ ability to upregulate prefrontal activity, it would have been useful to include transfer trials in which participants have to upregulate frontal activity without any direct feedback of their hemodynamic response. The absence of feedback during regulation trials can support a conclusion on how good a person got a feel for the actual brain state and how to modulate it.

Near-infrared spectroscopy data recorded during the letter n-back task indicated the use of different strategies for the condition with higher working memory load (2-back) relative to the condition with low working memory load (1-back). As anticipated, prior to neurofeedback training, in the 1-back task we found activation in PFC which is part of the working memory network (Baddeley, 2003; Owen et al., 2005; Ayaz et al., 2012; Fishburn et al., 2014; Herff et al., 2014). In contrast, in the 2-back condition, language associated areas tended to be more strongly activated indicating a more pronounced use of verbal strategies in the high load condition of our verbal working memory task. For such tasks, executive processes as well as the storage and recall of verbal material are required and verbal storage tasks in turn activate speech areas (Smith et al., 1996; Smith and Jonides, 1999; Baddeley, 2003).

Following eight sessions of neurofeedback, these activation patterns showed some changes, especially in the 2-back condition where activation within the above-mentioned language associated areas was now markedly reduced. These results might be indicative of a more efficient use of cognitive resources after the training, which would be especially noteworthy in the absence of systematic feedback-related learning effects as discussed above. Yet, these results have to be interpreted with caution as the present study lacks a control group and therefore the observed effects could also be due to simple time effects, repetition effects, automation or practice. Also, despite partly medium to strong effect sizes, the reported results did not withstand correction for multiple statistical testing in our small sample of healthy subjects. This might also be attributable to the relatively large brain region that was targeted by our neurofeedback protocol. However, to impact working memory, the upregulation possibly should have been more focused on a specific brain region (e.g., dorsolateral PFC) that is known to be involved in working memory task performance rather than covering a broad area of PFC.

In the long run, the aim is to conduct NIRS neurofeedback also in psychiatric patients which may benefit from this promising method. For this purpose, we propose some adjustments of the present training setup. First, for patients it might take longer to learn neural self-regulation and therefore the amount of sessions should be increased even though so far no standard has been established. First evidence suggests that 12 sessions provide an appropriate timeframe to learn to self-regulate hemodynamic activity (Marx et al., 2014). On average, EEG neurofeedback protocols applied in patient groups involve a total number of 25–40 sessions that take place one to two times per week (Moriyama et al., 2012; Mayer et al., 2013). This amount is cut down to 1–10 sessions with fMRI feedback (Sulzer et al., 2013) offering an apparently more time-efficient training approach, possibly due to advantages that go back to the method’s higher spatial resolution. NIRS is based on hemodynamic responses as well and is also characterized by a higher spatial resolution compared to EEG. Therefore, also for NIRS feedback, a smaller amount of required sessions can be expected which could already be confirmed by the NIRS feedback study in pediatric ADHD (Marx et al., 2014). Furthermore, the ideal interval between training sessions to ensure learning and enduring effects beyond the training phase is unknown. The issue remains whether learning success eventuates faster over the course of sessions in relatively close succession like in the present study or with a more distributed (lower) training frequency. A second future improvement of the currently employed protocol concerns the visualization of the feedback itself that should be as intuitive as possible without needless distracting factors (e.g., information about activation in channels that aren’t immediately relevant for the targeted feedback signal). Consequently, exclusively ROI activity should be fed back to the participants to reduce cognitive load. Thirdly, to ensure that patients specifically learn to regulate the target brain region, an adequate online artifact correction should be implemented for a clinical trial. This is to avoid general arousal effects all over the brain or artifacts for example induced by breathing or muscle activity. In summary, when taking into account a customization of these particular critical factors, NIRS neurofeedback could be applied also in a clinical context.

### Limitations

The current study was conducted as a proof-of-concept study with the primary objective to shed light on learning processes, the impact of strategies on individual learning as well as different learning types in NIRS neurofeedback. Therefore, only a small sample was recruited and no control group was included. However, despite the small sample size of 13, effect sizes reveal relatively robust effects.

## Conclusion

Despite the above-mentioned limitations the results of this study are promising. In the future, NIRS neurofeedback could be a time-effective and thereby more economic tool in (complementary) neuropsychiatric therapy. After some adjustments of the current setup, prefrontal NIRS feedback could also be utilized in different patient groups exhibiting alterations of PFC function, such as children or adults suffering from ADHD.

## Ethical Standards

The authors assert that all procedure contributing to this work comply with the ethical standards of the relevant national and institutional committees on human experimentation and with the Helsinki Declaration of 1975, as revised in 2008.

## Author Contributions

BB drafted the manuscript and analyzed the data. US participated in the design of the study and contributed to the manuscript. AF participated in the design of the study and contributed to the manuscript. A-CE conceived of the study, participated in its design and coordination, and helped to draft the manuscript. All authors read and approved the final manuscript. All authors agree to be accountable for all aspects of the work in ensuring that questions related to the accuracy or integrity of any part of the work are appropriately investigated and resolved.

## Conflict of Interest Statement

US was paid for public speaking by Novartis, Medice, Neuroconn, the German Society for Biofeedback and Akademie König und Müller. Where applicable, the above mentioned authors declare that the present work is unrelated to the above mentioned grants and relationships. All the other authors declare that the research was conducted in the absence of any commercial or financial relationships that could be construed as a potential conflict of interest.

## Abbreviations

ADHD: attention deficit-/hyperactivity disorder
BCI: brain computer interface
BOLD: blood oxygen level dependent
EEG: electroencephalography
fMRI: functional magnetic resonance imaging
Fpz: frontopolar central position in 10–20 system for electrode placement
HHb: deoxygenated hemoglobin
MNI: Montreal Neurological Institute
NIRS: near-infrared spectroscopy
O_2_Hb: oxygenated hemoglobin
ROI: region of interest
T3: left temporal position in 10–20 system for electrode placement
T4: right temporal position in 10–20 system for electrode placement
